# Supplementary material for: Three Novel Zn-Based Coordination Polymers: Synthesis, Structure, and Effective Detection of Al3+ and S2− Ions
Source: Molecules. 2020 Jan 17;25(2):382. doi: 10.3390/molecules25020382 (PMC7024230; doi:10.3390/molecules25020382)
Supplement: Supplementary file 1 [file molecules-25-00382-s001.pdf]

Three Novel Zn-Based Coordination Polymers: Synthesis, Structure, and Effective Detection of Al^{3+} and S^{2-} Ions

Yuna Wang [1,2,†], Xiaofeng Zhang [1,2,†], Yanru Zhao [1,2], Suoshu Zhang [1,2], Shifen Li [1,2], Lei Jia [1,2], Lin Du [1,2,*] and Qihua Zhao [1,2,*]

[1] School of Chemical Science and Technology Pharmacy, Yunnan University, Kunming 650091, China; wyn123456LL@163.com (Y.W.); zxf123456LL@163.com (X.Z.); zyr123456LL@163.com (Y.Z.); zss123456@163.com (S.Z.); lsf123456LL@163.com (S.L.); jl123456LLL@163.com (L.J.)

[2] Key Laboratory of Medicinal Chemistry for Natural Resource Education Ministry, Yunnan University, Kunming 650091, China

* Correspondence: lindu@ynu.edu.cn (L.D); qhzhao@ynu.edu.cn (Q.Z.); Tel.: +86-871-6503-5640 (L.D.)

† These authors contributed equally to this work.

Received: 27 December 2019; Accepted: 12 January 2020; Published: 17 January 2020

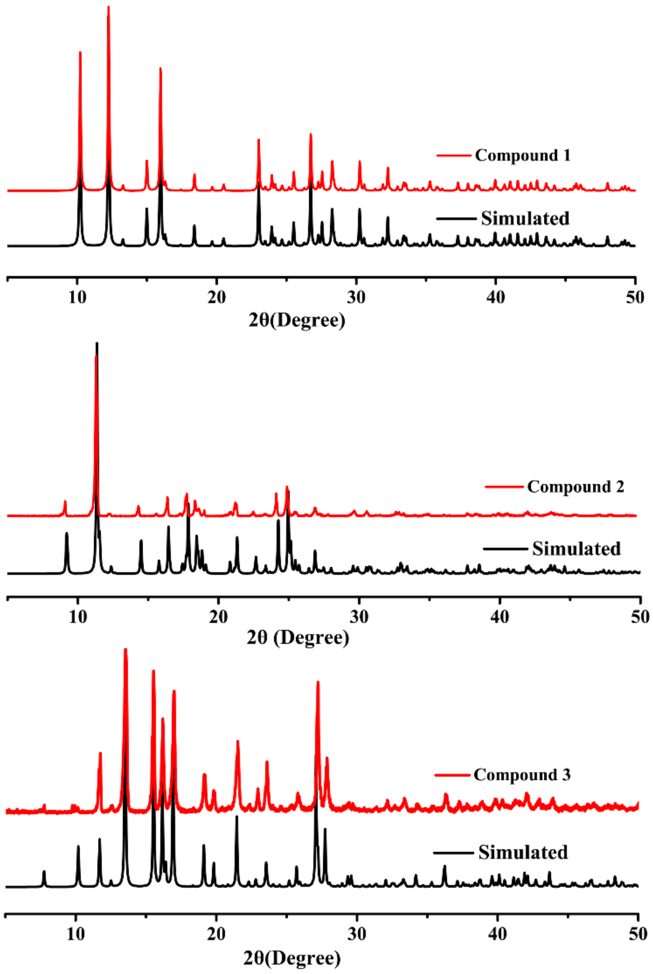

Figure S1. Powder diffraction (PXRD) of three compounds

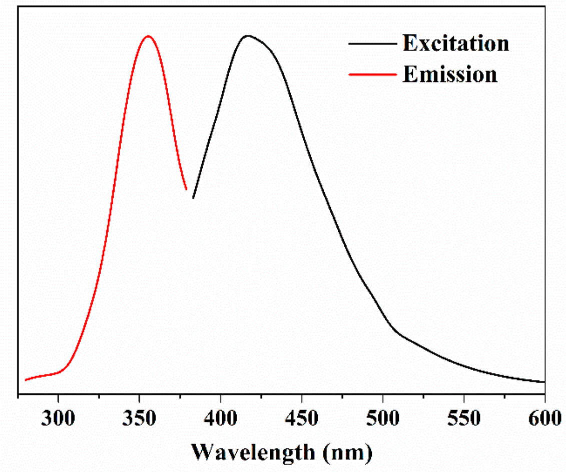

Figure S2. Fluorescence excitation and emission spectra of 4,4′-bpy in solid state.

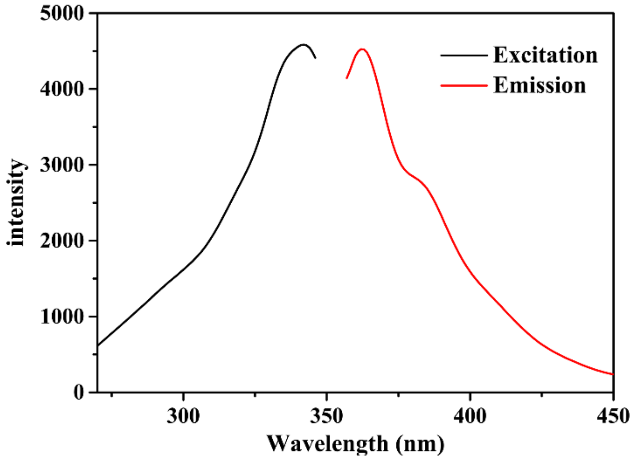

Figure S3. Fluorescence excitation and emission spectra of bpe ligand in solid state.

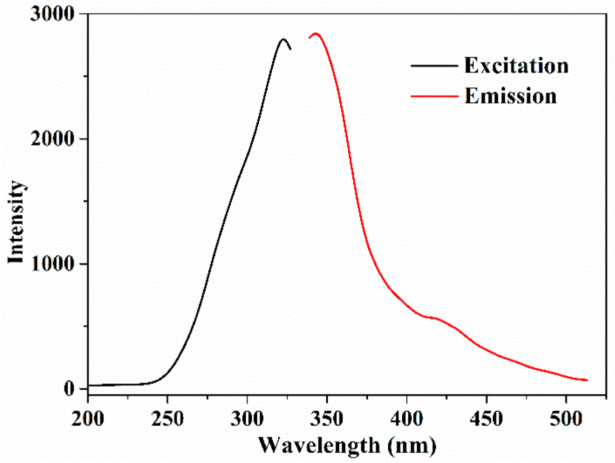

Figure S4. Fluorescence excitation and emission spectra of compound **1** in solid state.

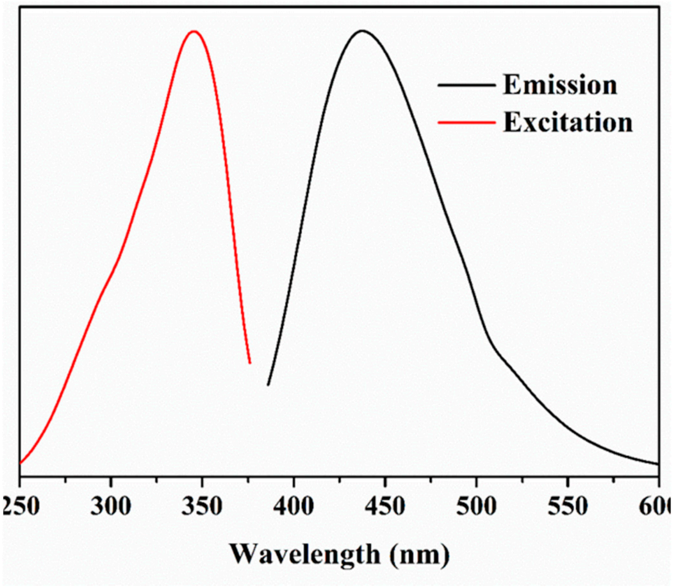

Figure S5. Fluorescence excitation and emission spectra of compound **2** in solid state.

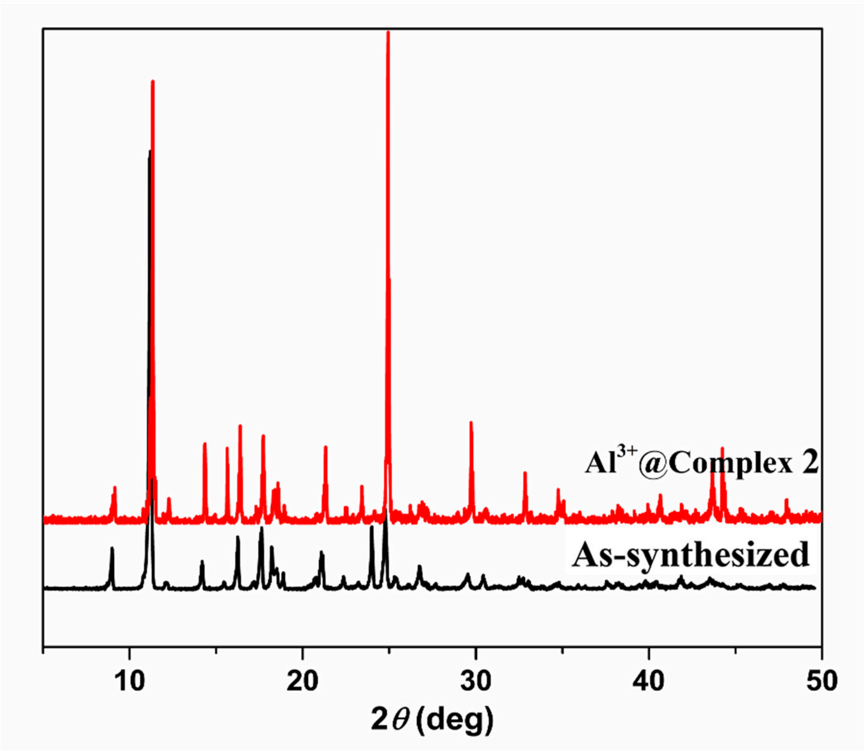

Figure S6. Powder X-ray pattern of Al^{3+}@compound **2** and As synthesized **2**.

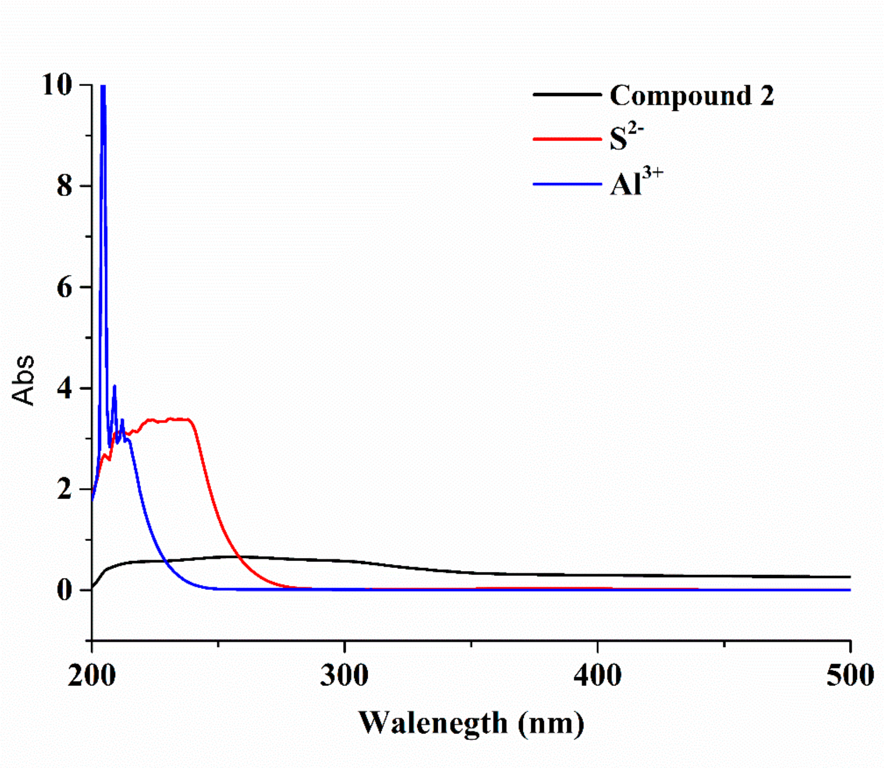

Figure S7. The ultravilet spectrum of the compound **2**, S^{2-} and Al^{3+} in ethanol solutions.

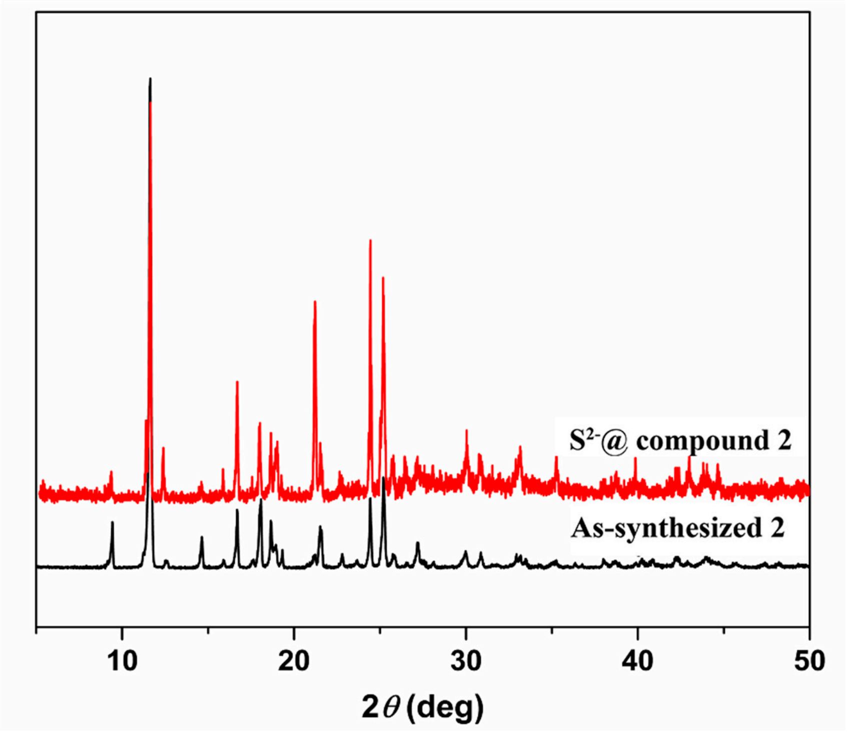

Figure S8. Powder X-ray pattern of S^{2-}@compound **2** and As synthesized **2**.

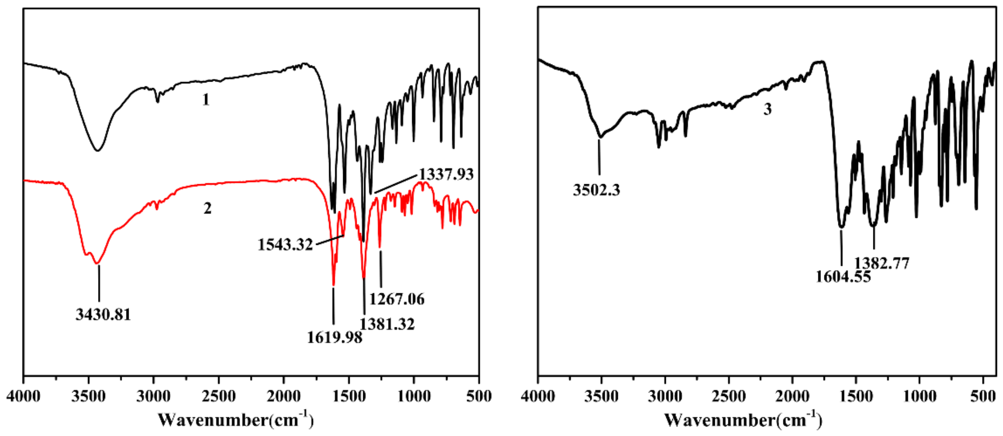

Figure S9. Infrared spectra of compounds.

Table S1 Selected bond lengths (Å) and angles (°) of compound **1**.

Zn1—O1^{1}	1.958 (4)	O1^{1}—Zn1—O4^{2}	105.83(15)
Zn1—O4^{2}	1.997 (4)	O1^{1}—Zn1—O2	109.01(17)
Zn1—O2	2.008 (4)	O4^{2}—Zn1—O2	93.44(16)
Zn1—O4^{3}	2.016 (4)	O1^{1}—Zn1—O4^{3}	101.14 (16)
Zn1—O5^{2}	2.482 (4)	O4^{2}—Zn1—O4^{3}	144.1(2)
O2—Zn1—O4^{3}	99.73(16)	O1^{1}—Zn1—O5^{2}	87.87(15)
O4^{2}—Zn1—O5^{2}	73.69(14)	O2—Zn1—O5^{2}	161.32(15)
O4^{3}—Zn1—O5^{2}	84.26(14)		

Symmetry codes for **1**: (1) *x*+1/2, *y*, -*z*+1/2; (2) *x*, -*y*+1/2, *z*-1/2; (3) *x*+1/2, -*y*+1/2, -*z*+1; (iv) *x*-1/2, *y*, -*z*+1/2; (v) *x*, -*y*+1/2, *z*+1/2; (vi) *x*-1/2, -*y*+1/2, -*z*+1.

Table S2 Selected bond lengths (Å) and angles (°) of compound **2**.

Zn(1)—O(3)^{1}	1.991(4)	O(3)^{1}—Zn(1)—N(1)	106.74(14)
Zn(1)—N(1)	2.024(4)	O(1)—Zn(1)—O(3)^{1}	106.17(13)
Zn(1)—O(1)	1.948(3)	O(1)—Zn(1)—O(5)	104.56(15)
Zn(1)—O(5)	2.055(3)	O(1)—Zn(1)—N(1)	138.76(15)
O(3)^{1}—Zn(1)—O(5)	94.69(13)	N(1)—Zn(1)—O(5)	96.80(15)

Symmetry codes for **2**: (1) *x*, -*y*+1, *z*-1/2, (2) *x*, 1-*y*, 1/2+*z*; (3) 1-*x*, 2-*y*, 1-*z*.

Table S3 Selected bond lengths (Å) and angles (°) of compound **3**.

Zn(1)—O(1)	1.934(3)	O(1)^{1}—Zn(1)—N(1)	106.92(15)
Zn(1)—O(1)^{1}	1.934(3)	O(1)^{1}—Zn(1)—N(1)^{1}	100.17(14)
Zn(1)—N(1)^{1}	2.059(3)	O(1)—Zn(1)—N(1)	100.17(14)
Zn(1)—N(1)	2.059(3)	N(1)^{1}—Zn(1)—N(1)	106.92 (15)
O(1)—Zn1—O1^{1}	134.0(2)	O(1)—Zn(1)—N(1)^{1}	106.4(2)

Symmetry codes for **3**: (1) *x*, -*y*+1/2, -*z*+1/2.
